# Targeting AKT-Dependent Regulation of Antioxidant Defense Sensitizes AKT-E17K Expressing Cancer Cells to Ionizing Radiation

**DOI:** 10.3389/fonc.2022.920017

**Published:** 2022-07-08

**Authors:** Isabell Goetting, Safa Larafa, Katharina Eul, Mikhail Kunin, Burkhard Jakob, Johann Matschke, Verena Jendrossek

**Affiliations:** ^1^ Institute of Cell Biology (Cancer Research), University Hospital Essen, Essen, Germany; ^2^ Department of Biophysics, GSI Helmholtzzentrum für Schwerionenforschung, Darmstadt, Germany; ^3^ Department of Biology, Technical University of Darmstadt, Darmstadt, Germany

**Keywords:** AKT, radioresistance, antioxidant defense, glycolysis, hexokinase 2, glutathione synthase, glutathione reductase

## Abstract

Aberrant activation of the phosphatidyl-inositol-3-kinase/protein kinase B (AKT) pathway has clinical relevance to radiation resistance, but the underlying mechanisms are incompletely understood. Protection against reactive oxygen species (ROS) plays an emerging role in the regulation of cell survival upon irradiation. AKT-dependent signaling participates in the regulation of cellular antioxidant defense. Here, we were interested to explore a yet unknown role of aberrant activation of AKT in regulating antioxidant defense in response to IR and associated radiation resistance.

We combined genetic and pharmacologic approaches to study how aberrant activation of AKT impacts cell metabolism, antioxidant defense, and radiosensitivity. Therefore, we used TRAMPC1 (TrC1) prostate cancer cells overexpressing the clinically relevant AKT-variant AKT-E17K with increased AKT activity or wildtype AKT (AKT-WT) and analyzed the consequences of direct AKT inhibition (MK2206) and inhibition of AKT-dependent metabolic enzymes on the levels of cellular ROS, antioxidant capacity, metabolic state, short-term and long-term survival without and with irradiation.

TrC1 cells expressing the clinically relevant AKT1-E17K variant were characterized by improved antioxidant defense compared to TrC1 AKT-WT cells and this was associated with increased radiation resistance. The underlying mechanisms involved AKT-dependent direct and indirect regulation of cellular levels of reduced glutathione (GSH). Pharmacologic inhibition of specific AKT-dependent metabolic enzymes supporting defense against oxidative stress, e.g., inhibition of glutathione synthase and glutathione reductase, improved eradication of clonogenic tumor cells, particularly of TrC1 cells overexpressing AKT-E17K.

We conclude that improved capacity of TrC1 AKT-E17K cells to balance antioxidant defense with provision of energy and other metabolites upon irradiation compared to TrC1 AKT-WT cells contributes to their increased radiation resistance. Our findings on the importance of glutathione *de novo* synthesis and glutathione regeneration for radiation resistance of TrC1 AKT-E17K cells offer novel perspectives for improving radiosensitivity in cancer cells with aberrant AKT activity by combining IR with inhibitors targeting AKT-dependent regulation of GSH provision.

## Introduction

Alterations in cell metabolism as a result from the activation of oncogenic pathways or the inactivation of tumor suppressor genes are a common characteristic of cancer cells (1, 2). Enhanced macromolecular synthesis is required to fuel rapid proliferation, whilst maintaining energy and redox balance (3). High metabolic activity results in pronounced cellular stress, e.g. through generation of reactive oxygen species (ROS) (4). Therefore, cancer cells upregulate cellular antioxidant defense to avoid lethal oxidative damage to cellular macromolecules (proteins, DNA, lipids) (5) and this process gains increasing importance with cancer progression (6). Importantly, metabolic adaptations enable cancer cells not only to survive environmental stress, but also enhance their capacity to cope with oxidative stress induced by cancer therapy and thereby to escape from genotoxic or targeted cancer therapy (7–9).

Radiotherapy (RT) is commonly used to treat cancer. Herein, ionizing radiation (IR) acts as local oxidizing agent that induces direct damage to the DNA including potentially lethal DNA double strand breaks (DSBs) (10–14). Moreover, radiolysis of water results in the formation of ROS and indirect damage to the DNA and other macromolecules, as well as organelle function (4). However, cells have evolved a complex DNA damage response (DDR) aimed at cellular protection from the lethal effects of DNA damage and to allow for DNA damage repair, or to induce cell inactivation or death in case of excessive DNA damage, respectively (15). Herein, repair pathways such as classical non-homologous end-joining (c-NHEJ), homologous recombination repair (HRR), alternative end-joining, or single strand annealing contribute to the repair of lethal DSBs [for more details (10, 13, 16)]. Other DNA repair pathways such as base excision repair (BER) help to protect cells from oxidative DNA damage (17). In addition, cells dispose of efficient antioxidant systems that support defense against ROS: These include ROS-detoxifying proteins like superoxide dismutase 1 (SOD1) and catalase (CAT), metabolic pathways that generate reduction equivalents needed to regulate the dithiol/bisulfide balance, and the glutaredoxin and thioredoxin systems that generate reduced forms of glutathione (GSH) and thioredoxin, respectively (18, 19). So far, the production of reduction equivalents to sustain antioxidant defense has been targeted in radiotherapy by inhibiting glycolysis-dependent production of nicotinamide adenine dinucleotide phosphate (NADPH) or glutaminolysis-dependent GSH generation, resulting in increased radiosensitivity (8, 20, 21).

Of note, cancer cells with high levels of GSH or high activity of glycolysis, pentose phosphate pathway (PPP) or glutaminolysis, experience an important advantage to dampen ROS levels and to escape the cytotoxic effects of RT, opening opportunities for targeted therapeutic intervention (8, 21, 22). It is tempting to speculate that oncogene-associated metabolic reprogramming allows cancer cells balance provision of NADPH for biosynthetic processes and for the regeneration of GSH in support of antioxidant defense under stress conditions (9, 23, 24).

In this context, activation of phosphatidyl-inositol-3-kinase (PI3K)/protein kinase B (AKT) has been associated with high GSH levels and ROS defense (25, 26). The PI3K/AKT pathway is one of the most frequently deregulated pathways in cancer cells and has been associated with cancer progression and resistance to genotoxic therapies (27–31). Oncogenic AKT signaling impacts multiple aspects of malignant behavior including cancer metabolic reprogramming and stress resistance (27, 32).

Own previous work revealed that cells with overexpression of the clinically relevant PH-domain mutant AKT1-E17K with aberrant activation of AKT accelerated DSB repair and improved the survival of TRAMPC1 prostate cancer cells (TrC1) upon IR (33). In contrast, the phosphorylation-deficient T308A/S473A (TASA) mutant (AKT1-TASA) had opposite effects (34). Of note, treatment with the AKT inhibitor MK2206 restored radiosensitivity in TrC1 cells expressing the resistance-promoting AKT1-E17K or AKT1-T308D/S473D (TDSD) (33). These findings indicate that aberrant activation of AKT promotes increased radiation resistance, presumably by phosphorylation-mediated regulation of effector proteins with impact on radiosensitivity, e.g., in DSB repair, or their transcriptional control (30, 31).

AKT is also involved in the regulation of cell metabolism, for example cellular antioxidant defense, and might thereby impact the cellular radiation response to IR: In fact, AKT regulates the early steps of glycolysis and the production of antioxidants like NADPH and GSH (27, 31). These regulatory processes involve post-translational regulation of the enzymatic activity of proteins at key metabolic nodes, or indirect regulation by transcriptional control of glycolytic, glutaminolytic, mitochondrial or lipid metabolic enzymes (27, 31). So far, the role of AKT-mediated metabolic regulation for cancer cell radioresistance is largely unknown.

We gained particular interest to study the role of AKT-dependent regulation of antioxidant defense and to explore the relevance of these processes for increased radiation resistance in cancer cells with aberrant activation of AKT. We used our established genetic model in TrC1 prostate cancer cells expressing either AKT1-WT or the clinically relevant activation associated AKT1-E17K mutant and pharmacologic approaches to explore how cancer cell-specific aberrant AKT activation and the activity of AKT-regulated metabolic pathways impact metabolic state and antioxidant defense at the basal state as well as changes in cellular levels of GSH, NADPH, and ROS, short-term and long-term survival upon irradiation.

Herein, we focused on the role of AKT-dependent direct regulation of metabolic enzymes involved in the regulation of glycolysis, generation of NADPH, novel synthesis or regeneration of GSH. For example, AKT phosphorylates and activates hexokinase 2 (HK2) (35). Furthermore, AKT is involved in the transcriptional regulation of glutathione synthase (GS) and γ-glutamylcysteine synthetase (γ-GCS), two enzymes that control key steps in the novel synthesis of GSH from cysteine, glycine and glutamate; AKT also regulates glutathione reductase (GR), the critical enzyme for the regeneration of reduced GSH from its oxidized form GSSG *via* the oxidation of NADPH to NADP^+^ (18, 19).

A deeper understanding of AKT’s role in the regulation of molecules and pathways that are critical to cell survival upon irradiation is required to develop effective strategies for radiation response modulation and to explore their potential for tumor-specific radiosensitization.

## Results

### Aberrant AKT Activation in TrC1 AKT-E17K Cells Is Associated With Increased Levels of NADPH and Improved Defense Against ROS

Hyperactive AKT is associated with increased radiation resistance (33, 36). Here, we used a genetic approach to study the impact of aberrant AKT activity on the antioxidative capacity and its contribution to the enhanced radioresistance of TrC1 cells overexpressing the clinically relevant AKT-E17K mutant compared to TrC1 cells overexpressing AKT-WT (**Figures S1D–G**). To study the relevance of AKT-dependent effects we treated the cells with the specific AKT-inhibitor MK2206 (37) (**Figure S2**) to access the AKT-regulated antioxidant defense capacity upon IR in our AKT-WT and activated AKT-E17K mutant TrC1 isogenic cell line models.

**Figure 1:**
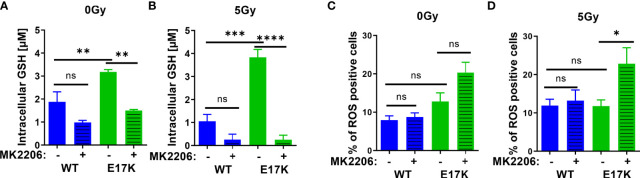
Expression of AKT-E17K is associated with increased levels of reduced cellular antioxidants and improved defense against ROS. TrC1 AKT-E17K and AKT-WT cells were treated with the AKT inhibitor MK2206 (4 µM) 1 h prior 5 Gy radiation and then analyzed 12 h after IR treatment or without IR treatment. GSH levels were analyzed using a luminescence-based assay upon indicated treatments at 12 h after IR **(A–B)**. ROS levels were determined by DHE using flow cytometry 24 h after non-irradiated controls **(C)** and after irradiation with 5 Gy **(D)**. Mean values and standard error of the mean (SEM) were scaled for data from at least 3 independent biological experiments with * p ≤ 0.05, ** p ≤ 0.01, *** p ≤ 0.001, **** p ≤ 0.0001, ns p > 0.05 using two-way ANOVA followed by Tukey’s multiple comparison test.

First, we measured the abundance of reduced glutathione species (GSH) and the influence of active AKT on GSH levels upon irradiation without or with additional treatment with the AKT-inhibitor MK2206. Untreated TrC1 cells expressing active AKT-E17K displayed significantly higher GSH levels than TrC1 cells overexpressing AKT-WT (**Figure 1A**). Irradiation with a single dose of 5 Gy led to a pronounced and sustained reduction of GSH that was still visible 12 h after irradiation, whereas GSH levels of irradiated TrC1 AKT-E17K cells at 12 h after irradiation were similar to untreated control cells (**Figure 1B**). Importantly, treatment with MK2206 efficiently reduced GSH levels in unirradiated AKT-E17K cells (**Figure 1A**) and significantly abrogated GSH levels 12 h after irradiation in TrC1 AKT-E17K cells (**Figures 1A, B**). These results corroborated the suggested regulation of cellular GSH by AKT (25) (**Figures 1A, B**).

Further, analyzing a time-dependent regulation of GSH after IR demonstrated reduced GSH 1 h post IR in TrC1 AKT-WT and AKT-E17K cells and subsequent recovery of GSH between 6-24 h after IR (**Figure S1A**). Recovery of GSH levels was faster in TrC1 AKT-E17K cells compared to AKT-WT cells, and largely delayed by MK2206 treatment in both cell types (**Figure S1A**). These findings support AKT-dependent effects on GSH levels and their recovery.

To gain insight into potential consequences of AKT-E17K expression and MK2206-mediated AKT inhibition on the formation of cellular ROS in untreated and irradiated TrC1 cells, we also determined the amount of ROS-positive cells by flow cytometry using DHE staining. Enhanced ROS formation as a consequence of increased oncogenic signaling and a compensatory increase of cellular antioxidant defense have been described by others (38–40). In fact, we observed a trend to a higher amount of ROS-positive TrC1 AKT-E17K cells compared to AKT-WT cells without irradiation. Treatment with MK2206 did not significantly increase the amount of ROS-positive AKT-E17K and AKT-WT cells without irradiation (**Figure 1C**).

Furthermore, irradiation has been demonstrated to trigger the formation of ROS that damage cellular components, e.g., the DNA, and thereby contribute to the cytotoxic action of ionizing radiation (41, 42). Consequently, improved defense against cellular ROS by cellular antioxidant systems like glutathione supports resistance against the cytotoxic effects of ionizing radiation (8, 23, 43). Single dose irradiation with 5 Gy slightly increased the number of ROS positive AKT-WT cells compared to untreated cells but was without effect in AKT-E17K cells (**Figures 1C, D**). However, pretreatment with MK2206 significantly enhanced the amount of ROS-positive AKT-E17K mutant cells upon irradiation (**Figure 1D**). These data pointed to a role of AKT-E17K for the protection against radiation-induced ROS in these cells.

Finally, determination of acute cell death 48 h after MK2206 treatment revealed increased cell death levels in irradiated AKT-E17K cells (**Figures S1B, C**). Pretreatment with the synthetic GSH-derivate glutathione ethyl ester (GEE) significantly reduced ROS and cell death levels induced upon MK2206 treatment alone or in combination with IR in TrC1 Akt-E17K cells (**Figures S1D–G**). Moreover, determination of long-term survival by standard colony formation assays revealed an increased radioresistance of TrC1 AKT-E17K cells compared to AKT-WT cells (**Figures S1H–K**), corroborating own previous work (33). Additional pre-treatment with MK2206 16 h before IR resulted in a significantly reduced survival in both, TrC1 AKT-WT and AKT-E17K cells (**Figures S1H–K**) (33, 34).

So far, our data suggested that expression of AKT-E17K improves protection against ROS, presumably by increasing cellular antioxidant capacity, and thereby contributes to enhanced radioresistance of TrC1 AKT-E17K cells. However, the contribution of specific AKT-regulated processes to improved antioxidant defense was unclear.

### TrC1 AKT-E17K Mutant Cells Have a Higher Glycolytic Activity and Improved Recovery From Radiation-Induced Glycolytic Impairment

To scale the impact of the AKT-dependent regulation of glycolysis on antioxidant defense, first, glycolytic activity was measured in our AKT mutant model cells in a basal state and in response to IR, and second, the effects of inhibition of AKT target hexokinase 2 (HK2) were explored.

AKT impacts glycolytic function by regulating expression and phosphorylation of different target proteins (27, 31), such as phosphorylation of glucose by HK2, which mediates the first step of glycolysis. Of note, activation of glycolysis is also required to fuel glucose-6-phosphate into the PPP and to produce NADPH for glutathione regeneration. We thus hypothesized, that aberrant AKT activation in TrC1 AKT-E17K cells may ensure increased antioxidant defense by fueling glycolysis and PPP to produce NADPH. To gain insight in potential differences in the metabolic phenotype of AKT-WT and AKT-E17K cells we compared the basic energy metabolism of AKT-WT and AKT-E17K TrC1 cells by extracellular flux technology using the Seahorse Bioanalyzer.

**Figure 2:**
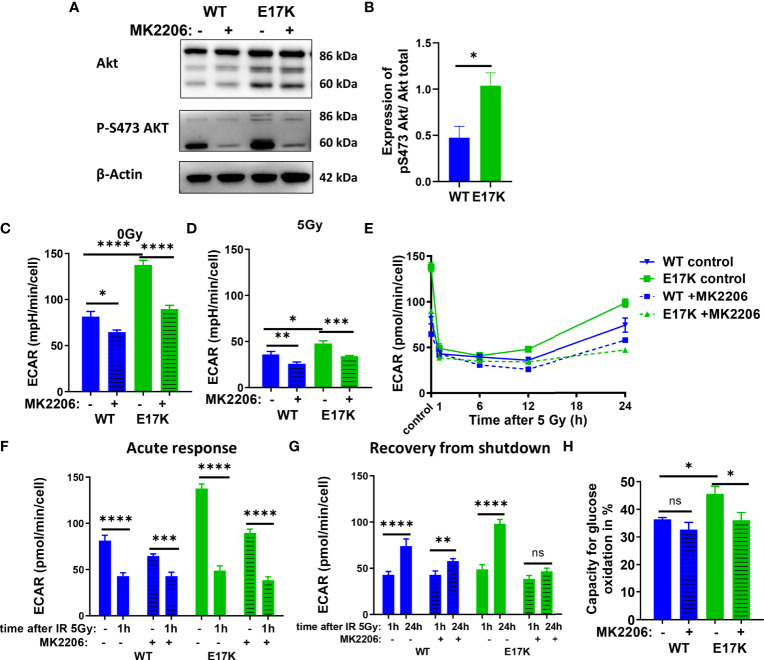
Glycolytic activity is increased in TrC1 AKT-E17K cells and supports recovery from metabolic inhibition induced by IR. **(A)** Akt phosphorylation on S473 for unirradiated TrC1 AKT-WT and AKT-E17K cells measured in western blot and normalized on total AKT expression and β-actin. Representative pictures are shown in **(A)** with quantification in **(B)**. Basal glycolytic activity of TrC1 AKT-WT and AKT-E17K cells at 0 Gy and 5 Gy with and without MK2206 (4 µM) treatment was measured in an extracellular flux assay (Seahorse technology) and extracellular acidification rate (ECAR) values are shown for 0 Gy and 12 h after 5 Gy **(C–D)**, time dependent regulation of basal glycolysis in the first 24 h after 5 Gy **(E)**, the acute response after radiation **(F)** and the recovery phase after IR **(G)**. In an extracellular flux assay (Seahorse technology) glucose capacity **(H)** was determined using the fuel flex assay. Mean values and standard error of the mean (SEM) were scaled for data from n=3 independent biological experiments **(A, B)** or n = 8-16 wells from 2 independent biological experiments **(C-H)** with * p ≤ 0.05, ** p ≤ 0.01, *** p ≤ 0.001, **** p ≤ 0.0001, ns p > 0.05 using standard unpaired t-test **(A, B)**, two-way ANOVA followed by Tukey’s multiple comparison test **(C–E, H)** or mixed effect analysis multiple comparison with Sidak’s correction **(F-G)**.

TrC1 AKT-E17K cells were characterized by a significant increase in basal glycolytic activity as displayed by the extracellular acidification rate (ECAR) (**Figure 2C**) compared to AKT-WT cells. Although glycolytic rates were reduced in both cell types 12 h after irradiation with 5 Gy, glycolytic rate was still significantly higher in AKT-E17K cells (**Figure 2D**). Pre-treatment with the AKT inhibitor MK2206 (4 µM) significantly reduced glycolytic rates, particularly in AKT-E17K cells, pointing to AKT-dependent effects (**Figures 2C–E**). Interestingly, exposure to IR led to a rapid decline in glycolytic function 1 h after 5 Gy in both cell lines (**Figures 2F, G**) that was followed by a recovery of glycolytic function within 12-24 h after IR. These observations corroborate our previous results on a radiation-induced inhibition of metabolic processes (OxPhos and glycolysis) (44). Of note, the active AKT-E17K variant accelerated whereas AKT inhibition by MK2206 impaired recovery of glycolytic function until 24 h after irradiation (**Figures 2E, G**). These observations support AKT-dependent effects on the regulation of glycolytic activity upon IR treatment (**Figures 2F, H**). Furthermore, expression of AKT-E17K also increased compensatory glycolysis, the additional glycolytic activation after electron transport chain (ETC) inhibition, highlighting the importance of glycolytic activity upon IR treatment (**Figures S3A–C**).

Additionally, the capacity to oxidize glucose on the mitochondria was measured using Seahorse Bioanalyzer and the fuel flex assay revealing increased capacity to fuel mitochondrial respiration by oxidation of glucose in TrC1 AKT-E17K cells compared to AKT-WT cells (**Figure 2H**). Of note, MK2206 treatment decreased the capacity of AKT-E17K cells to oxidize glucose to the levels of the TrC1 AKT-WT cells (**Figure 2H**) emphasizing AKT’s impact on glucose metabolism. Furthermore, TrC1 AKT-E17K cells experience an AKT-dependent increase in glucose uptake, thereby allowing these cells to increase their glucose metabolism (data not shown). In contrast, no differences between TrC1 AKT-E17K mutant and AKT-WT cells with or without additional MK2206 treatment on glutamine capacity were observed (**Figure S3D**).

Last, to determine the impact of AKT-dependent regulation of glycolysis on NADPH production we used Fluorescence Lifetime Imaging Microscopy (FLIM) technology to compare NADPH/NADH ratio in AKT-E17K and AKT-WT TrC1 in untreated controls and upon irradiation with 5 Gy with and without additional treatment with the AKT-inhibitor MK2206 (**Figure S3E, F**). Cells overexpressing AKT-E17K or AKT-WT differed neither in the basal NADPH/NADH ratio (untreated cells) nor in NADPH/NADH ratio determined 12 h after irradiation. However, AKT-inhibition by MK2206 had a pronounced effect on the NADPH/NADH ratio, both in the basal state without irradiation and 12 h after irradiation with a single dose of 5Gy (**Figures S3E, F**). Interestingly, the MK2206 effect on the NADPH/NADH ratio was less pronounced in the unirradiated AKT-E17K cells compared to the AKT-WT cells (**Figures S3E**); furthermore, MK2206-treatment revealed increased mean fluorescent lifetime (tm) of NADPH and NADH species without and with IR, particularly in the untreated TrC1 AKT-E17K cells, hinting to an increase of NADH levels as a potential cause for the decreased NADPH/NADH ratio (**Figures S3G-I**).

These data indicate that overexpression of AKT-E17K upregulates glycolytic function in TrC1 cells and supports recovery of glycolytic function upon irradiation, with potential benefit for cell survival. The increased glycolytic activity in turn did not increase NADPH reductive species in TrC1 AKT mutant cells.

### Inhibition of Glycolysis by the Hexokinase 2 (HK2)-Inhibitor 2-Deoxyglucose (2DG) Impairs Glycolytic Activity and NADPH Production

Our previous data revealed that overexpression of AKT-E17K increased glycolytic activity and associated radioresistance in an AKT-dependent manner. We were therefore interested if inhibition of the AKT target protein hexokinase 2 (HK2) which catalyzes the rate limiting step of glycolysis by using the HK2 inhibitor 2-Deoxyglucose (2DG) would mimic the effects of the AKT inhibitor MK2206 on the antioxidant defense and survival after IR.

**Figure 3:**
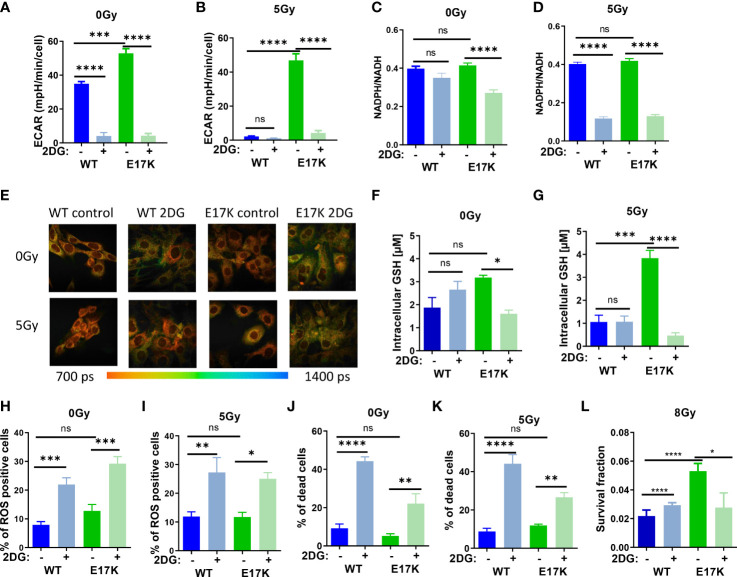
Inhibition of glycolytic activity by treatment with 2-deoxyglucose (2DG) affects antioxidant levels and enhances radiosensitivity. TrC1 AKT-WT and AKT-E17K cells were pre-treated for 1 h with the solvent control or the hexokinase inhibitor 2DG (10 mM) and subsequently exposed to irradiation with 0 Gy (controls) or 5 Gy as indicated. 12 h after the irradiation time point we determined **(A, B)** extracellular acidification rate (ECAR) by an extracellular flux assay; **(C, D)** NADPH/NADH ratio using FLIM measurement; and **(F, G)** GSH levels using a luminescence assay. Photomicrographs in **(E)** depict FLIM pictures for 0 Gy and 12 h after 5 Gy showing tm with representative pictures of one representative experiment out of three. ROS levels were measured in flow cytometry using DHE-staining for **(H)** 0 Gy and **(I)** 24 h after 5 Gy. Bar diagrams in **(J, K)** depict cell death levels detected by flow cytometry using PI-staining. **(L)** Long term survival of TrC1 AKT-WT and AKT-E17K cells pre-treated with 0 or 10 mM 2DG and subsequently irradiated with 0-8 Gy was determined by indirect colony formation assays; data depict the surviving fraction upon irradiation with 8 Gy. Mean values and standard error of the mean (SEM) were scaled for data from at least 3 independent biological experiments with * p ≤ 0.05, ** p ≤ 0.01, *** p ≤ 0.001, **** p ≤ 0.0001, ns p > 0.05 using two-way ANOVA followed by Tukey’s multiple comparison test **(A, B, F–L)** or one way ANOVA followed by multiple test **(C, D)**.

As expected, treatment with 2DG almost abrogated glycolytic activity in AKT-WT and AKT-E17K cells at baseline (without irradiation) and after irradiation with 5 Gy (**Figures 3A, B**) and thereby also lowered mitochondrial respiration measured in the extracellular flux assay (**Figures S4 A, B**).

Furthermore, 2DG treatment significantly decreased NADPH/NADH ratios measured by FLIM in irradiated AKT-WT and AKT-E17K expressing TrC1 cells (**Figures 3C, D**) whereas effects on NADPH/NADH ratio at baseline were only observed in TrC1 AKT-E17K cells. Similar to the effects of MK2206 treatment (**Figure S1A-B**), 2DG treatment also increased the mean fluorescence of reductive NADPH and NADH species, presumably due to an increase of NADH species (**Figures 3E**, **S4C, D**).

Next, we investigated how 2DG treatment alters the levels of ROS and acute cell death. As depicted in **Figures 2H–K**, 2DG treatment led to a significant increase in the proportion of ROS-producing cells (**Figures 3H, I**) and cell death (**Figures 3J, K**) in TrC1 AKT-WT and AKT-E17K cells, irrespective of additional irradiation. Of note, TrC1 AKT-WT cells turned out to be more sensitive to 2DG-induced acute cell death than AKT-E17K cells. However, when analyzing the effect of 2DG treatment on clonogenic survival upon irradiation, we observed that 2DG did almost not alter clonogenic survival of TrC1 AKT-WT cells whereas radiosensitivity of TrC1 AKT-E17K cells was slightly decreased, at least at a higher single irradiation dose (8 Gy) (**Figure 3L**). Instead, no significant reduction of the survival fraction was observed upon 2DG treatment in combination with lower doses of IR (**Figures S4E–I**).

Interestingly, 2DG treatment acutely lowered the initially higher levels of GSH, as illustrated before (**Figure 1A**), only in TrC1 AKT-E17K cells (**Figure 3F**), but not in TrC1 AKT-WT cells (**Figure 3F**). The reduction of GSH levels upon 2DG treatment was even more pronounced when AKT-E17K cells where exposed to irradiation (**Figure 3G**). To explore, if the effect of 2DG on survival of irradiated TrC1 AKT-E17K cells was due to the associated reduction in GSH, we measured long-term survival of TrC1 AKT-WT and AKT-E17K cells upon treatment with 2DG without supplementation with GEE and with additional irradiation. Of note, supplementation with GEE rescued the survival of AKT-E17K cells upon treatment with 2DG and irradiation with 8 Gy (**Figures S4E–H**), suggesting a role of GSH for the survival of irradiated cells with impaired glycolytic activity.

These findings highlight a connection between glycolysis and antioxidant defense and suggest that higher glycolytic activity of TrC1 AKT-E17K cells supports their antioxidant capacity, rendering the cells vulnerable to 2DG mediated inhibition of glycolysis and associated production of reduction equivalents and GSH.

### Inhibition of the Glutathione Synthesis Reduces GSH Levels and Radiosensitizes TrC1 AKT-E17K Cells

**Figure 4:**
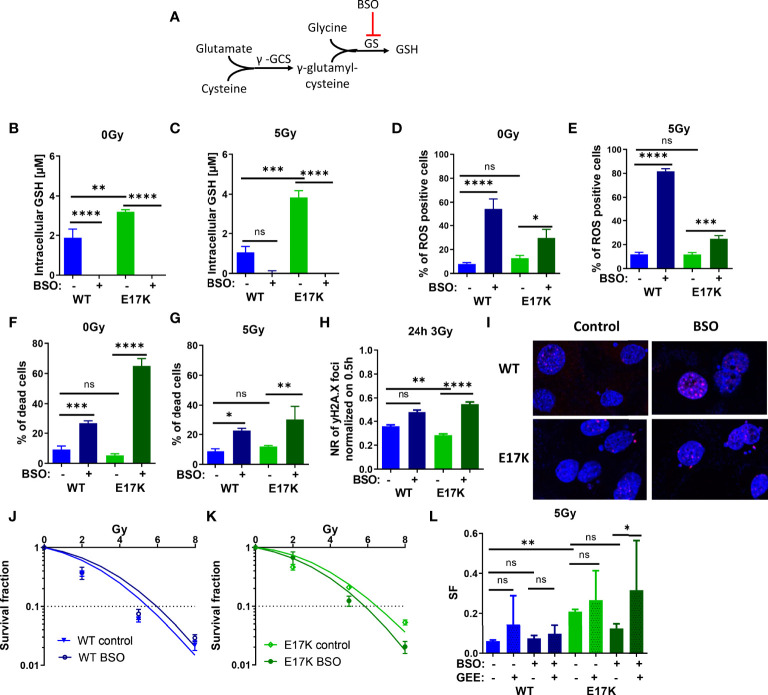
Pharmacologic inhibition of glutathione synthetase (GS) enhances sensitivity of TrC1 AKT-E17K cells to ionizing radiation (IR) by disturbing antioxidant defense. **(A)** Schematic representation of the GSH generation pathways *via* γ-glutamylcysteine synthetase (γ-GCS) and GS and its inhibition by buthionine sulfoximin (BSO) on the GS. Different parameters for radiation response were quantified for TrC1 AKT-E17K and AKT-WT cells treated with the GS inhibitor BSO (200 µM) 1 h before 5 Gy radiation or without IR. GSH levels were analyzed in a luminescence-based assay 12 h after 0 Gy **(B)** and 5 Gy **(C)** treatments. A flow cytometry-based analysis of ROS-positive cells 24 h after IR **(E)** compared to non-irradiated controls **(D)** was used for ROS quantification. Cell death levels were determined in non-irradiated 0 Gy controls **(F)** and 48 h after 5 Gy **(G)** treatment. The number of γH2A.X foci 30 min controls was analyzed *via* fluorescence microscopy 24 h after 3 Gy and BSO (200 µM) treatment in comparison with the controls (H-I). In an indirect colony formation assay, TrC1 AKT-WT and AKT-E17K cells were treated with BSO (20 µM) and irradiated with doses from 0-8 Gy and SF was plotted dose dependently in **(J-K)** and in a bar chart for 5 Gy treatment with additional glutathione ethyl ester (GEE, 4 mM) supplementation 1 h prior IR (L). Mean values and standard error of the mean (SEM) were scaled for data from at least 3 independent biological experiments with * p ≤ 0.05, ** p ≤ 0.01, *** p ≤ 0.001, **** p ≤ 0.0001, ns p > 0.05 using two-way ANOVA followed by Tukey’s multiple comparison test **(A-G, J-L)** or one way ANOVA followed by multiple test **(H)**.

Next, we focused on targeting the generation of GSH, which is regulated downstream of Akt *via* nuclear factor erythroid 2-related factor 2 (Nrf2)-dependent expression of the involved genes. Indeed, Nrf2 expression was upregulated in TrC1 Akt-E17K cells 6 h after 5 Gy (**Figure S5**). Therefore, we investigated if downstream targeting of glutathione synthesis by pharmacologic inhibition of glutathione synthase (GS) using the established GS-inhibitor buthionine sulfoximine (BSO) would impact GSH levels in an AKT-dependent manner (45, 46) (**Figure 4A**). In fact, inhibition of GS significantly reduced GSH levels in AKT-WT and AKT-E17K cells with and without IR (**Figures 4B, C**). The increased GSH levels in TrC1 AKT-E17K compared to AKT WT cells were already described in 2.1.

Furthermore, inhibition of GS by BSO significantly increased ROS levels 24 h after treatment in TrC1 AKT-WT and AKT-E17K cell lines (**Figures 4D, E**) and enhanced cell death in AKT-WT and Akt-E17K mutant cell lines irrespective of additional irradiation with a dose of 5 Gy after 48 h (**Figures 4F, G**).

Further, we analyzed if the enhanced ROS levels after inhibition of GS impact the levels of IR-induced DNA damage. Therefore, we quantified the kinetics of formation and resolution of radiation induced DNA damage foci using immunofluorescent staining with the DSB marker γH2A.X. Interestingly, inhibition of GS by BSO significantly enhanced the residual number of radiation-induced γH2A.X foci 24 h irradiation with 3 Gy in TrC1 AKT-E17K cells (**Figures 4H**), whereas γH2A.X foci 30 min after 3 Gy were not influenced by BSO (**Figure S7A**).

We further analyzed the impact of AKT-regulated GSH-synthesis for cancer cell survival upon IR-treatment. Therefore, we directly pre-treated the AKT-WT and AKT-E17K mutant TrC1 cells with BSO before irradiation and measured the long-term survival upon irradiation with different radiation doses (0 Gy, 2 Gy, 5 Gy, 8 Gy) by using a colony formation assay. Inhibition of GS by BSO significantly reduced the survival fraction (SF) of TrC1 AKT-E17K cells after IR whereas no significant effect was observed in AKT-WT cells (**Figures 4J–L**). These data revealed a strong reliance of AKT-E17K cells on GSH-synthesis for the survival upon IR. Notably, additional treatment with GEE reversed at least partly BSO-induced radiosensitization of AKT-E17K cells (**Figures 4L**, **S6A**).

Our findings demonstrate that pharmacologic inhibition of GS is suited to radiosensitize active AKT-mutant cells (E17K) and suggest that increased GSH synthesis contributes to increased radioresistance of TrC1 AKT-E17K cells.

### Inhibition of Glutathione Reductase Reduces GSH Levels and Radiosensitizes TrC1 AKT-E17K Cells to IR

**Figure 5:**
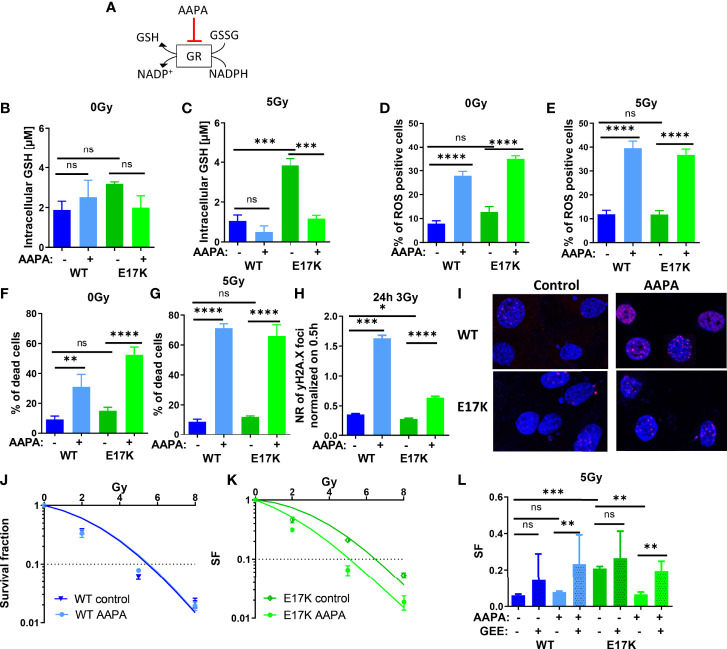
Pharmacologic inhibition of glutathione reductase (GR) disturbs cellular antioxidant defense and sensitizes TrC1 E17K cells to ionizing radiation (IR). **(A)** Schematic representation of the mechanism of reduced glutathione (GSH) generation and the action of the inhibitor of GR AAPA. The effects on TrC1 AKT-E17K and AKT-WT cells of the inhibition of GR by AAPA (40 µM) treatment 1 h before 5 Gy or 0 Gy were analyzed. GSH levels were quantified in a luminescence-based assay 12 h after IR **(C)** or without IR **(B)**. In flow cytometry ROS levels were determined by DHE-staining 24 h after respective treatments **(D, E)** whereas cell death levels were accessed by quantification of the % of PI-positive cells 48 h after treatment **(F, G)**. The number of γH2A.X foci normalized on 30 min controls was measured 24 h after 3 Gy and AAPA (40 µM) treatment in comparison with the controls **(H, I)**. TrC1 AKT-WT and AKT-E17K cells were treated with AAPA (4 µM) 1 h prior to irradiation with doses from 0-8 Gy and colony formation assay was performed determining survival fractions in doses-dependent curves **(J, K)** and in a bar chart for 5 Gy treatment with additional glutathione ethyl ester (GEE, 4 mM) supplementation 1 h prior IR **(L)**. Mean values and standard error of the mean (SEM) were scaled for data from at least 3 independent biological experiments with * p ≤ 0.05, ** p ≤ 0.01, *** p ≤ 0.001, **** p ≤ 0.0001, ns p > 0.05 using two-way ANOVA followed by Tukey’s multiple comparison test **(A-G, J-L)** or one way ANOVA followed by multiple test **(H)**.

Besides regulating GSH synthesis, AKT is also involved in GSH regeneration by regulating the expression of glutathione reductase (GR) (5, 27). GR converts GSSG to 2 GSH using NADPH oxidation to NADP^+^, thereby regulating the equilibration of oxidative and reductive species (as illustrated schematically in **Figure 5A**). We therefore speculated that AKT-mediated upregulation of GR may indirectly contribute to the increased radioresistance observed in AKT-E17K cancer cells. In order to gain insight into a potential role of GR in the cellular radiation response we treated the cancer cells with the documented GR inhibitor 2-acetylamino-3-[4-(2-acetylamino-2- carboxyethylsulfanylthio-carbonylamino)-phenylthiocarbamoylsulfanyl]propionic acid (AAPA) (47, 48) (**Figure 5A**).

Here, inhibition of GR by AAPA significantly reduced GSH levels in TrC1 AKT-E17K cells 12 h after IR with 5 Gy (**Figures 5B**). AAPA treatment exerted neither a significant effect on GSH levels in TrC1 AKT-WT cells nor on GSH levels in unirradiated cells (**Figures 5B, C**). Furthermore, AAPA treatment also significantly increased ROS levels (**Figures 5D, E**) and cell death levels (**Figures 3F, G**) in AKT-WT and AKT-E17K cells irrespective of irradiation. Similar to the results obtained with inhibition of GSH-synthesis, GR also enhanced the levels of residual γH2A.X foci 24 h after 3 Gy in both, AKT-WT and E17K mutant cells (**Figures 5H, I**). However, long-term survival of AKT-WT cells was not affected by AAPA-treatment (**Figures 5J, L**). In contrast, inhibition of GR by AAPA significantly sensitized AKT-E17K cells to radiation-induced eradication of clonogenic tumor cells (**Figures 5K, L**). Of note, exogenous GEE supplementation was able to rescue TrC1 AKT-E17K cells from the radiosensitizing effects of AAPA-mediated GR-inhibition (**Figures 5L**, **S6B**). These data indicate that GR-dependent GSH regeneration was essential for the survival of irradiated TrC1 AKT-E17K cells.

Altogether our data demonstrate a role of AKT-E17K in improving both, GSH synthesis and GSH regeneration. Moreover, AKT enhanced glycolytic activity after IR thereby fueling energy metabolism and antioxidant defense thereby impacting radiation response *via* different nodes.

## Discussion

The survival kinase AKT has documented relevance for intrinsic cancer cell resistance to radiotherapy. Here, we demonstrate that expression of the clinically relevant activation-associated AKT mutant protein AKT-E17K increased cellular antioxidant capacity, particularly cellular GSH levels, and thereby enhanced radioresistance of TrC1 prostate cancer cells. Moreover, we dissect metabolic processes and relevant metabolic enzymes underpinning AKT-E17K dependent upregulation of GSH and associated radioresistance: In addition to the direct AKT target protein HK2, which regulates a rate-limiting step of glycolysis (35), we identified a critical role of GS and GR for AKT-E17K-dependent upregulation of cellular antioxidant defense and associated radioresistance. Both proteins are regulated by the transcription factor Nrf2 downstream of its activation by AKT, oxidative stress, or other stressors (24). Importantly, pharmacologic inhibition of GS or GR sensitized TrC1 AKT-E17K cells to IR and was sufficient to overcome AKT-E17K-mediated increased radioresistance. Instead, pharmacologic inhibition of HK2 by 2DG treatment impaired activity of glycolysis which is associated with PPP. Glycolytic inhibition disturbed AKT-E17K-dependent antioxidant capacity by metabolic rewiring. Taken together, deciphering AKT-dependent metabolic processes driving radioresistance cancer cell allowed to discover new and effective combination therapies that may be suited to improve the outcome of treatments involving radiotherapy.

In more detail, overexpression of AKT-E17K enhanced oxidative stress in TrC1 cells and this was associated with higher glycolytic rate, a rise in cellular levels of reduced GSH, and increased radioresistance. Pharmacologic inhibition of AKT by treatment with the AKT inhibitor MK2206 significantly decreased GSH levels in untreated TrC1 AKT-E17K cells and almost abrogated GSH levels 12 h after irradiation, and this was associated with a significantly increase in radiosensitivity. These findings corroborate suggested effects of AKT on the regulation of the important cellular antioxidant GSH (25, 49).

Activation of AKT by oxidative stress and AKT-dependent regulation of antioxidant defense in cancer have already been reported by others (49, 50). However, here we demonstrate in addition that the clinically relevant AKT-E17K mutant variant enhances cellular GSH levels in the basal state and has relevance to enhanced radiation resistance of AKT-E17K expressing TrC1 cells. These observations are reminiscent on the role of increased antioxidant capacity (GSH levels) allowing to balance ROS levels and associated radioresistance induced by metabolic adaptation of cancer cells to chronic cycling hypoxia (8, 9).

Considering the role of AKT-E17K on the formation of reduction equivalents in the form of NADPH we found that AKT-E17K modulates cellular antioxidant capacity rather on the level of reduced GSH than *via* modulation of NADPH levels. For example, MK2206 treatment decreased NADPH/NADH ratio, whereas the fluorescence lifetime (tm) was increased upon MK2206 treatment alone or in combination with IR, presumably due to an increase in NADH species. Here, increased mean fluorescence lifetime could represent a switch from free to bound NADPH and NADH species described to experience a longer lifetime (51), which might be caused by the metabolic stress induced by AKT inhibition. However, we cannot exclude that the pronounced increase of NADH compared to NADPH upon AKT inhibition might be the result from a reduced activity of complex I within the electron transport chain (52) or of the AKT target protein NADK. Reduced NADK activity would reduce generation of NADPH by NADH phosphorylation, whilst promoting NADH accumulation (53, 54). Instead, we did not observe significant changes in NADPH levels and therefore assume that due to its importance for energy metabolism cells tend to keep NADPH levels constant.

AKT is a key regulator of various cellular processes including cell proliferation and survival (47, 50). We thus hypothesized, that increased proliferation and metabolic activity may enhance dependency of AKT-E17K TrC1 cells on cellular antioxidant defense and associated metabolic pathways. In fact, TrC1 AKT-E17K cells displayed higher glycolytic activity compared to the TrC1 AKT-WT cells. Furthermore, we observed a higher dependency of TrC1 AKT-E17K cells on oxidation of glucose on the mitochondria compared to AKT-WT cells, which was reduced by Akt inhibitor MK2206. Thus, our data confirm that TrC1 AKT-E17K cells can use glucose to fuel mitochondrial metabolism and thus might experience increased metabolic flexibility ensuring increased survival compared to AKT-WT cells. Interestingly, exposure to IR led to an acute impairment of mitochondrial respiration and glycolytic activity that was followed by a time-dependent recovery. These observations corroborate similar findings described in our recent study (44). There we concluded that high glycolytic activity is needed to fuel needs in reduction equivalents, energy and metabolites for DNA repair and antioxidant defense during the acute radiation-induced mitochondrial shutdown (44). Of note, TrC1 AKT-E17K cells had a higher capacity to resume glycolytic activity after the radiation-induced acute impairment of glycolysis and oxidative phosphorylation, whereas treatment with the AKT-inhibitor MK2206 attenuated the recovery of glycolytic function of irradiated cells. We thus speculate that high compensatory glycolysis in AKT-E17K cells may help the cells to balance antioxidant defense and energy needs in support of survival upon irradiation.

To challenge our hypothesis, we subsequently investigated if high glycolytic activity and dependency on glucose may offer opportunities for counteracting radioresistance of TrC1 AKT-E17K cells. HK2 is a direct target protein of AKT that catalyzes the first step of glycolysis and thereby also influences PPP (31, 35). Herein, the HK2 inhibitor 2DG has been described as a potent inhibitor of glycolysis and efficient radiosensitizer, at least for highly glycolytic tumors (21, 55). We therefore chose 2DG treatment to investigate if targeting would allow to counteract two AKT-regulated processes with assumed relevance to radioresistance, namely compensatory glycolysis and antioxidant defense. In our hands, 2DG treatment reduced glycolytic activity and this was associated with an increase of ROS-positive cells and acute cell death in AKT-WT and AKT-E17K cells. Moreover, 2DG treatment significantly decreased GSH levels in TrC1 AKT-E17K cells. However, 2DG had only minor effects on clonogenic survival upon irradiation: a significant radiosensitization was only observed in AKT-E17K cells when 2DG treatment was combined with a single high-dose irradiation with 8 Gy. The minor effect of glycolysis inhibition compared to AKT inhibition on radiosensitivity of AKT-E17K cells revealed that the effects of AKT-E17K radiosensitivity go beyond the regulation of glycolytic activity.

Multiple reports revealed links between increased antioxidant capacity and glycolytic activity of cancer cells as a mechanism underlying therapy resistance (8, 21, 22, 43, 56). The minor effects of 2DG on cancer cell radiosensitivity compared to AKT-inhibition observed in the present study may be due to the indirect effects of glycolysis inhibition on the cellular antioxidant capacity, e.g. by generation of NADPH in the PPP. Under these conditions the highly interconnected metabolic pathways will allow cancer cells treated with 2DG to switch to alternative pathways for generation of NADPH and GSH in support of survival under oxidative stress (57, 58). Along these lines, the delayed upregulation of GSH observed seven days after 2DG treatment may contribute to the minor radiosensitizing effect of 2DG treatment in AKT-E17K cells.

The above data corroborate effects of oncogenic AKT in the regulation of basal glycolytic activity without irradiation and of compensatory glycolysis in irradiated with potential impact on radiosensitivity. Improved capacity to activate compensatory glycolysis upon irradiation may help the cancer cells to balance energy metabolism and generation of metabolites for DNA DSB repair with metabolic pathways fueling antioxidant defense. However, our findings also indicate that the effects of AKT-E17K on radiosensitivity of TrC1 cells go beyond its role in regulating glycolytic activity. Herein, AKT dependent regulation of mitochondrial respiration, glutaminolysis and lipid metabolism may impact outcome of RT (31, 59), and we cannot exclude that these pathways impact radiation resistance of AKT-E17K expressing TrC1 cells.

However, since our results implicated that AKT-E17K modulates cellular antioxidant capacity rather on the level of reduced GSH than *via* modulation of NADPH levels we focused on establishing potential roles of AKT-E17K in regulating GSH provision at the level of glutathione synthesis and glutathione regeneration: In fact, inhibition of GS and GR, two critical enzymes acting downstream of the AKT target Nrf2 (31, 60), by using BSO and AAPA, efficiently reduced GSH levels and increased the fraction of ROS-positive cells, the number of residual γH2A.X foci, and acute cell death in irradiated AKT-WT and AKT-E17K cells. BSO treatment efficiently reduced GSH levels in AKT-WT and AKT-E17K cells without and with irradiation, suggesting that GSH synthesis is a major mechanism for GSH provision. Instead, AAPA treatment decreased GSH levels only in AKT-E17K cells and in combination with IR. These findings suggest that glutathione regeneration may become important in AKT-E17K mutant cells for maintaining high GSH levels upon exposure to radiation-induced oxidative stress. We presume, that GR inhibition might impair the function of certain proteins and processes like the de-glutathionylation of proteins leading to further impairment of antioxidant defense and high ROS levels independently of GSH regulation (61, 62). Additionally, AAPA acts also on thioredoxin thereby impairing antioxidant capacity *via* the thioredoxin system (47).

Inhibition of GS and GR both enhanced eradication of irradiated clonogenic cells and thus radiosensitivity only in TrC1 AKT-E17K cells. These findings point to a reduced dependency of irradiated TrC1 AKT-WT cells on GSH for their survival. However, BSO or AAPA treatment both decreased the surviving fraction of AKT-E17K cells to the level of AKT-WT cells and were thus suited to overcome the survival advantage of AKT-E17K expressing cells upon RT. Even more important, the radiosensitizing effects of BSO and AAPA were rescued by the synthetic GSH-derivate GEE emphasizing the critical role of GSH-related effects for the survival of irradiated AKT-E17K cells. Overall, our data implicate a strong dependence of cancer cells expressing the active AKT-E17K mutant on their enhanced GSH levels for protection against ROS and cell survival, though the TrX system and ROS-detoxifying enzymes (SOD1, catalase) might still be relevant for the maintenance of antioxidant capacity in TrC1 AKT-E17K cells.

In summary, our data demonstrate that increased radioresistance of cancer cells expressing the clinically relevant activation-associated AKT mutant protein AKT-E17K involves multiple metabolic mechanisms: On the one hand, AKT-E17K promoted increased glycolytic activity at basal state and improved the capability of the cells for activating compensatory glycolysis upon irradiation. On the other hand, AKT-E17K enhanced the levels of the cellular antioxidant GSH by promoting both, GSH novel synthesis and GSH regeneration. The increase in GSH levels was critical for coping with radiation-induced ROS and DNA damage and for supporting survival of clonogenic TrC1 AKT-E17K cells upon irradiation. In turn, disturbance of AKT-E17K-dependent antioxidant defense by pharmacologic inhibition of glutathione synthase, or glutathione reductase resulted in efficient radiosensitization of TrC1 AKT-E17K cells. Thus, deciphering oncogene-dependent metabolic processes promoting radioresistance is crucial for the discovery of new and effective combination therapies that may be suited to improve the outcome of treatments involving radiotherapy.

## Material and methods

### Cell Lines and Cell Culture

TrC1 murine prostate cancer cell lines had been stably transfected with pBABE-AKT1-WT-GFP-puro or pBABE-AKT1-E17K-GFP-puro by Sebastian Oeck as described earlier [23]. These cells were used as genetic models of TrC1 cells with enhanced or impaired activation state of the survival kinase AKT. Cells were cultured in DMEM medium including 1% penicillin/streptomycin, 10% FCS (Biochrom AG), and 4 µg/ml puromycin.

### Irradiation and Drug Treatments

X-ray irradiation was performed at room temperature with X-RAD 320 X-Ray Biological Irradiator (Precision X-ray, North Branford, CT, USA) using 320 kV, 12.5 mA, a 1.65 mm aluminum filter and a dose rate of 3.40 Gy/min with the cells in a distance of 50 cm were or the X-ray generator ISO-DEBYEFLEXVOLT 3003 with an industrial X-ray tube 160 M1/10-55 with installed shutter (15b) operated at 35kV and 80mA (GE Sensing & Inspection Technologies GmbH & Co.KG, Ahrensburg, Germany). Control cells were kept at room temperature for the same period of time as their irradiated counterparts.

Cells were treated with AAPA (40 µM, Merck KGaA, Darmstadt, Germany), BSO (200 µM, Sigma-Aldrich, St. Louis, MO, USA), MK2206 (4 µM, Selleckchem, Houston, TX, USA) or 2DG (10 mM, Sigma-Aldrich, St. Louis, MO, USA) 1 h prior to IR. Rescue experiments (as described in (63)) were performed using 4 mM glutathione ethylester (GEE; Cayman Chemical, Ann Arbor, MI, USA) which was applied 1 h prior to IR. For long term colony formation assays, the drug concentration was reduced for AAPA to 4 µM and BSO 20 µM.

### Glutathione Measurement

For glutathione measurements the GSH/GSSG Glo assay (Promega GmbH, Walldorf, Germany) was used according to the manufacturers protocol allowing to determine oxidative and reductive glutathione species in cells *via* luminescence as previously described (43).

### Determination of ROS and Cell Death by Flow Cytometry

ROS can be determined using dihydroethidium (DHE) staining and cell death by propidium iodide (PI) staining the dead cells. Samples then can be measured using flow cytometry distinguishing stained and unstained cells. For that, cells were collected and stained in the dark with DHE (5 µM in PBS) for 30 min at 37 °C or PI (10 µg/ml in PBS) 30 min at room temperature (RT). Stained cells were measured with FACS Calibur, (Becton Dickinson, Heidelberg, Germany) or CytoFLEX S Flow Cytometer (Beckman Coulter GmbH, Krefeld, Germany). Data analysis was performed using the FlowJo software (FlowJo, LLC, Ashland, OR, USA) and CytExpert Software (Beckman, Brea, CA, USA). Cells were measured (>10,000 cells) and to determine ROS, % of ROS positive cells were distinguished from ROS negative cells due to their higher fluorescence of DHE and for cell death analysis, the fraction of dead cells (PI-positive population) was quantified.

### Determination of NADH, NADPH by Fluorescence Lifetime Imaging

For the analysis of NADH and NADPH levels, their blue autofluorescence upon UV-excitation can be used. FLIM additionally allows discriminating free and bound states (64). For FLIM experiments, cells were seeded in 3.5 cm glass bottom dishes (Greiner Bio-One, Frickenhausen, Germany) using DMEM culture media without phenol-red. 24 h later, cells were treated and irradiated with x-rays at the FLIM setup described in (65). Fluorescence lifetime of NADPH and NADH was measured using a Ti : Sa laser (Coherent Chameleon Ultra II, Dieburg, Germany) for 2-Photon excitation (720 nm; 80 MHz) at certain time points after IR including non-irradiated controls. The fluorescence lifetime decay was recorded for 90 sec for each field of view by using the Olympus IX71 microscope (Olympus Europa SE & CO. KG, Hamburg, Germany) coupled to a DCS-120 confocal FLIM System (Becker & Hickl GmbH, Berlin, Germany) operated by SPCM64 software (Becker & Hickl GmbH, Berlin, Germany). The fluorescence signal was detected behind a 458/64 nm bandpass filter using a non-descanned detection port and a hybrid bialkali photodetector HPM-100 (Becker & Hickl GmbH, Berlin, Germany). Fluorescence lifetime data were fitted using the incomplete multi-exponential model of the SPCimage software (Becker & Hickl GmbH, Berlin, Germany) with the fast decay time (t1) fixed to 350 ps (corresponding to the lifetime of free NAD(P)H), a binning of 3 and a threshold set to 30 photons. Raw images showing photon counts, fraction of free (a1) and bound species (a2), fluorescence decay time of bound species (t2) and quality of fit (chi2) were exported. With the use of the program ImageD (by David Eilenstein, https://github.com/DavidEilenstein/ImageD), cells were segmented and values were determined for each cell separately originating from the bright mitochondrial signal. NADPH/NADH ratio and fractions of oxidative/reductive species were calculated according to Blacker et al. (64). The mean fluorescence lifetime (tm) for each cell was calculated as follows: tm=a1 x t1 + a2 x t2.

### Determination of Metabolic Parameters by Extracellular Flux Assay

Metabolic analysis was performed using the Seahorse Xfe96 analyzer (Agilent Technologies, Santa Clara, CA, USA) measuring the oxygen consumption rate (OCR) and extracellular acidification rate (ECAR) of cells pre-treated with inhibitors and at certain time points after radiation. Measurements were performed in HEPES-buffered DMEM with 5 mM glucose and 1 mM glutamate, which was changed 1 h before the assay. The measurements were performed either by measuring basal OCR and ECAR or using the Glycolytic Rate Assay according to manufacturer’s protocol. The latter included the application of 0.5 µM rotenone and 0.5 µM antimycin A followed by 50 mM 2DG and 3 measurements of OCR and ECAR before and after each application. In the fuel flex test kit, the inhibitor of glutaminase (GLS1) BPTES (3 µM), the inhibitor of carnitine palmitoyl-transferase 1 A (CPT1A) etomoxir (4 µM) and the inhibitor of the mitochondrial pyruvate carrier (MPC) UK5099 (2 µM) were applied in different combinations according to manufacturer’s protocol to determine metabolic capacity and dependency. The determination of cell numbers was done with 30 µM Hoechst in PBS by measuring the fluorescence in a microplate reader. The assays were analyzed using the Wave 2.4 software (Seahorse Bioscience, Billerica, MA, USA).

### Determination of DSB *via* Immunofluorescence Staining for γH2A.X foci

Irradiated cells (3 Gy) were fixed (3 % PFA) and permeabilized (0.5 % Triton) for 15 min at specific time points (30 min, 1 h, 3 h, 6 h, 12 h, 24 h) followed by blocking with 0.2 % normal goat serum in PBS. Cells were stained with γH2A.X-AF647-conjugated antibody (1:100; both BD Biosciences, Franklin Lakes, NJ, USA) for 1 h at RT and nuclei with Hoechst (6 µM in PBS) for 5-10 min. After washing for 3x with PBS, samples were mounted with DAKO mounting medium (DAKO, Glostoup, Denmark) and stored in the dark at 4 °C. The Zeiss Axio Observer Z1 microscope (Carl Zeiss AG, Oberkochen, Germany) with ApoTome and 63er-oil objective was used to take fluorescence images. The analysis of the foci was performed using the focinator software (66) for minimum of 50 nuclei for each independent experiment. Repetitions of 3 biological independent experiments were performed.

### Colony Formation Assay

The colony formation assay was used to determine the influence of a treatment on the long-term survival of irradiated cancer cells after specific radiation doses compared to the clonogenic survival of untreated cells after IR as described previously in (43, 44). Due to strong effects of the used inhibitors (except MK2206), indirect plating of colonies was performed. Therefore, cells were treated with the specific inhibitor 1 h prior to irradiation with 0-8 Gy. After 24 h, cells were collected (0.05% Trypsin, 0.01 EDTA) and seeded in low densities (100-6400 cells/well) on 6-wells in inhibitor-free media (delayed plating). For the direct colony formation assay, cells were seeded in low densities (100-6400 cells/well) on 6-wells. After getting attached (8 h), cells were treated with MK2206 (4 µM) and irradiated after inhibitor incubation for 16 h. In both versions, cells were cultured in standard conditions for 7-9 days before quantifying colony numbers. Therefore, colonies were fixed with 3,7 % formaldehyde, washed with 70 % ethanol and stained with 0.05 % Coomassie brilliant blue 7-9 days after seeding. After manual counting of colony numbers, the plating efficiency and survival fraction were calculated as described in (67).

### Measurement of Protein Expression and Phosphorylation *via* Western Blot

Western Blot was used to scale the expression and phosphorylation of different proteins after IR or to control inhibitor efficiency. Cell pellets were collected and lysed using lysis buffer (50 mM Tris-HCl, 0.5 % NP-40 (Nonidet), 120 mM NaCl, 10 mM NaPP, 1 mM EDTA, 2 mM Na_3_VO_4_) including protease and phosphatase inhibitors for 1 h on ice. Protein concentrations were determined by Bradford reagent (Biomol GmbH, Hamburg, Germany). Then, concentrations were equilibrated, sodiumdodecylsulfate (SDS) sample buffer was added and samples were heated up to 95 °C for 5-10 min. 15-20 µg protein per sample were added on 12 % SDS polyacrylamide gels and ran for ca. 4 h at 130 V in SDS running buffer (25 mM Tris-HCl, 19,2 mM Glycine, 1 % SDS). Gels were blotted onto methanol-activated polyvinylidene fluoride (PVDF) membranes in Tris-glycine buffer (25 mM Tris-HCl, 19.2 mM glycine) for 105 min at 1 A. After blocking with 5 % milk powder in TBS-T (0.5 M Tris, 1.5 M NaCl, 1 % Tween-20) membranes were incubated with the primary antibodies (1:1000 in 5 % BSA in TBS-T, β-Actin 1:20000) overnight. Membranes were washed 3× with TBS-T and incubated for 1 h with horseradish peroxidase-conjugated secondary antibodies (1:2000 in 5 % BSA in TBS-T). Detection of antibody binding was performed in Fusion Solo Developing (Peqlab GmbH, Erlangen, Germany) device by Fusion software (Vilber Lourmat, Eberhardzell, Germany) using ECL™ Prime or Select detection reagents (Sigma-Aldrich, St. Louis, MO, USA). Antibodies were purchased from Cell Signaling (total Akt # 9272, pS473 Akt # 9271, anti-mouse-HRP conjugated, anti-rabbit-HRP conjugated), Thermofisher (Nrf2 PA5-27882) and Sigma Aldrich (β-Actin).

### Statistics

Statistical analysis was performed using GraphPad Prism 7.0 Software (GraphPad, La Jolla, California). Data from 3 independent biological experiments were used and mean value and standard error of the mean (SEM) calculated. Statistical significances were determined by t-test or one-way or two-way ANOVA. Meaning of the stars: * p ≤ 0.05, ** p ≤ 0.01, *** p ≤ 0.001, **** p ≤ 0.0001, ns p>0.05.

## Data Availability Statement

The original contributions presented in the study are included in the article/**Supplementary Material**. Further inquiries can be directed to the corresponding authors.

## Author Contributions

IG, JM, VJ and BJ designed the research. IG, JM and VJ wrote the manuscript. IG, SL and KE performed the experiments. IG, SL, KE, MK and BJ analyzed the results. IG, MK, BJ and JM validated and visualized the results. VJ, JM and BJ acquired the funding. All authors critically revised, edited, and approved the final version of the manuscript.

## Funding

This work was supported through funding by the German Research Council, DFG (GRK1739/2 to VJ, and MA 8970/1-1 to JM), the European Commission Horizon 2020 (Marie Skłodowska-Curie Innovative Training Network THERADNET; Grant Agreement No. 860245 (ITN THERADNET) to VJ and JM, the Federal Ministry of Education and Research (BMBF, 02NUK061B) to JM and BMBF 02NUK054A to BJ.

## Conflict of Interest

The authors declare that the research was conducted in the absence of any commercial or financial relationships that could be construed as a potential conflict of interest.

## Publisher’s Note

All claims expressed in this article are solely those of the authors and do not necessarily represent those of their affiliated organizations, or those of the publisher, the editors and the reviewers. Any product that may be evaluated in this article, or claim that may be made by its manufacturer, is not guaranteed or endorsed by the publisher.
