# Supplementary figures and images for: Targeting AKT-Dependent Regulation of Antioxidant Defense Sensitizes AKT-E17K Expressing Cancer Cells to Ionizing Radiation
Source: Front Oncol. 2022 Jul 8;12:920017. doi: 10.3389/fonc.2022.920017 (PMC9304891; doi:10.3389/fonc.2022.920017)

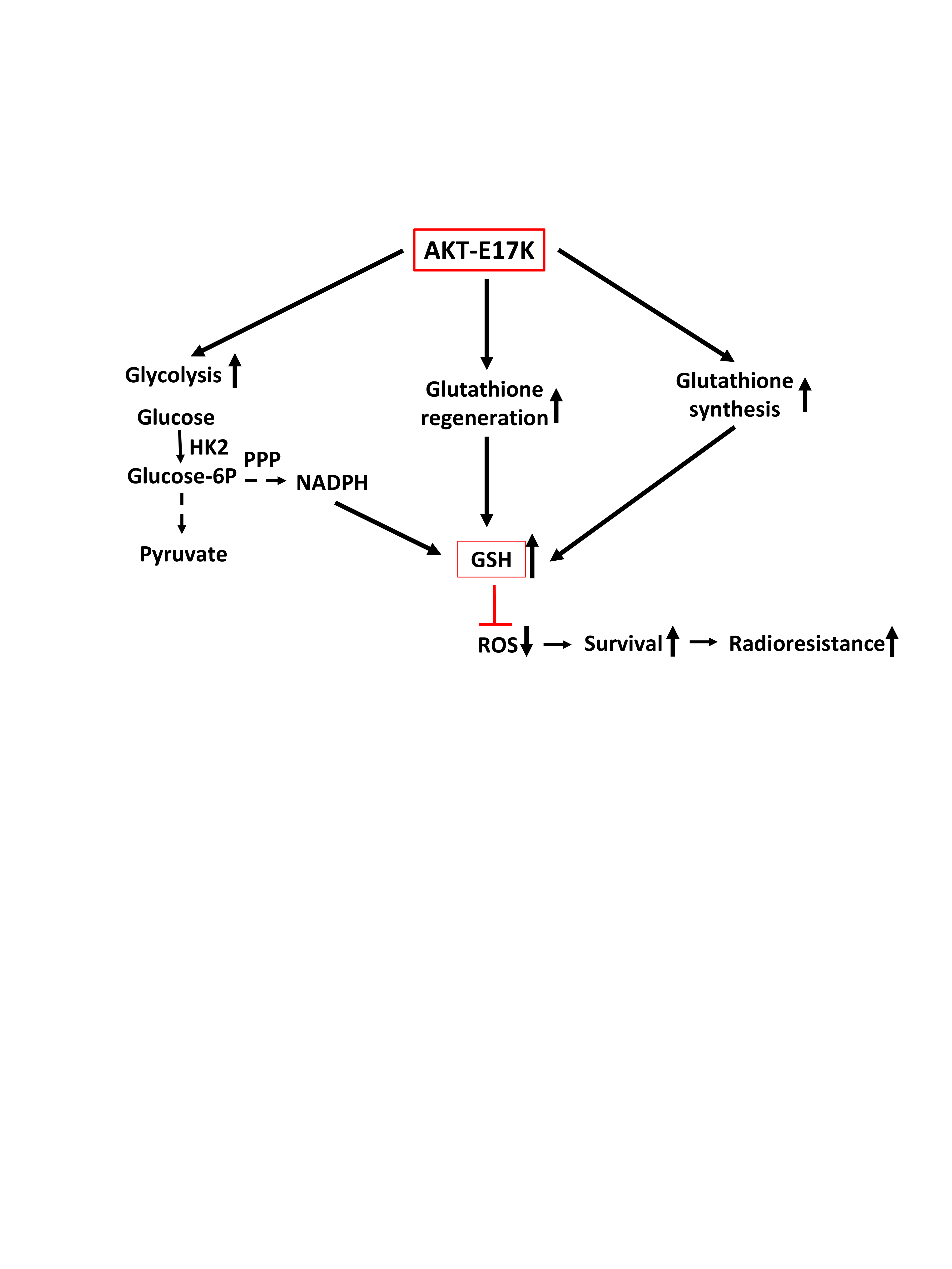

Supplement: Supplementary file 1 [file Image_1.tif]
