# Supplementary material for: Targeting AKT-Dependent Regulation of Antioxidant Defense Sensitizes AKT-E17K Expressing Cancer Cells to Ionizing Radiation
Source: Front Oncol. 2022 Jul 8;12:920017. doi: 10.3389/fonc.2022.920017 (PMC9304891; doi:10.3389/fonc.2022.920017)
Supplement: Supplementary file 2 [file DataSheet_1.docx]

Supplementary Material

# Supplementary Data

## Supplementary Figures


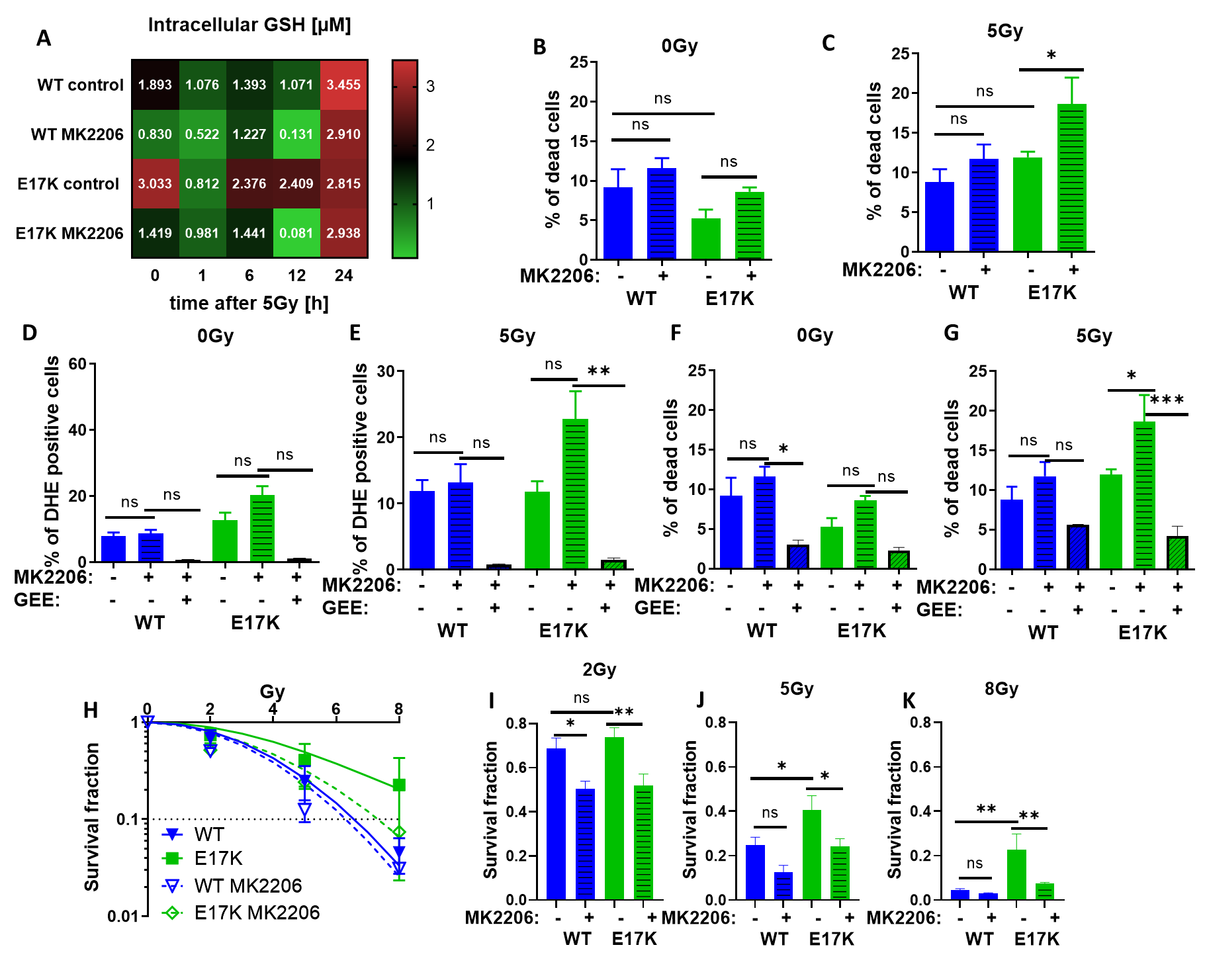


**Supplementary Figure 1.** **Pharmacologic AKT-inhibition affects time-dependent reconstitution of GSH levels and sensitizes TrC1 AKT-E17K cells to acute cell death and clonogenic cell death.** TrC1 AKT-E17K and AKT-WT cells were left untreated or treated with the AKT inhibitor MK2206 (4 µM) 1 h prior to irradiation with 0 or 5 Gy and analyzed 12 h after the irradiation time-point. (A) Time-dependent regulation of GSH levels after IR determined in a luminescence assay. Cell death levels were determined upon PI staining by using flow cytometry 48 h after IR with 5 Gy (C) compared to non-irradiated controls (B). TrC1 AKT-WT and AKT-E17K cells were treated with MK2206 (4 µM) and GEE (4 mM) 1 h prior IR with 5 Gy or left unirradiated. Flow cytometry was used to measure ROS using DHE (A-B) or cell death using PI (D-G). Clonogenic survival after radiation was measured for TrC1 AKT-WT and AKT-E17K pretreated with MK2206 (4 µM) 16 h prior IR by standard colony formation assays. (H) Dose-dependent survival curves and (I-K) bar charts for 2 Gy, 5 Gy and 8 Gy are displayed. Mean values and standard error of the mean (SEM) were scaled for data from at least 3 independent biological experiments with * p ≤0 .05, ** p ≤ 0.01, *** p ≤ 0.001, **** p ≤ 0.0001, ns p > 0.05 using two-way ANOVA followed by Tukey’s multiple comparison test.


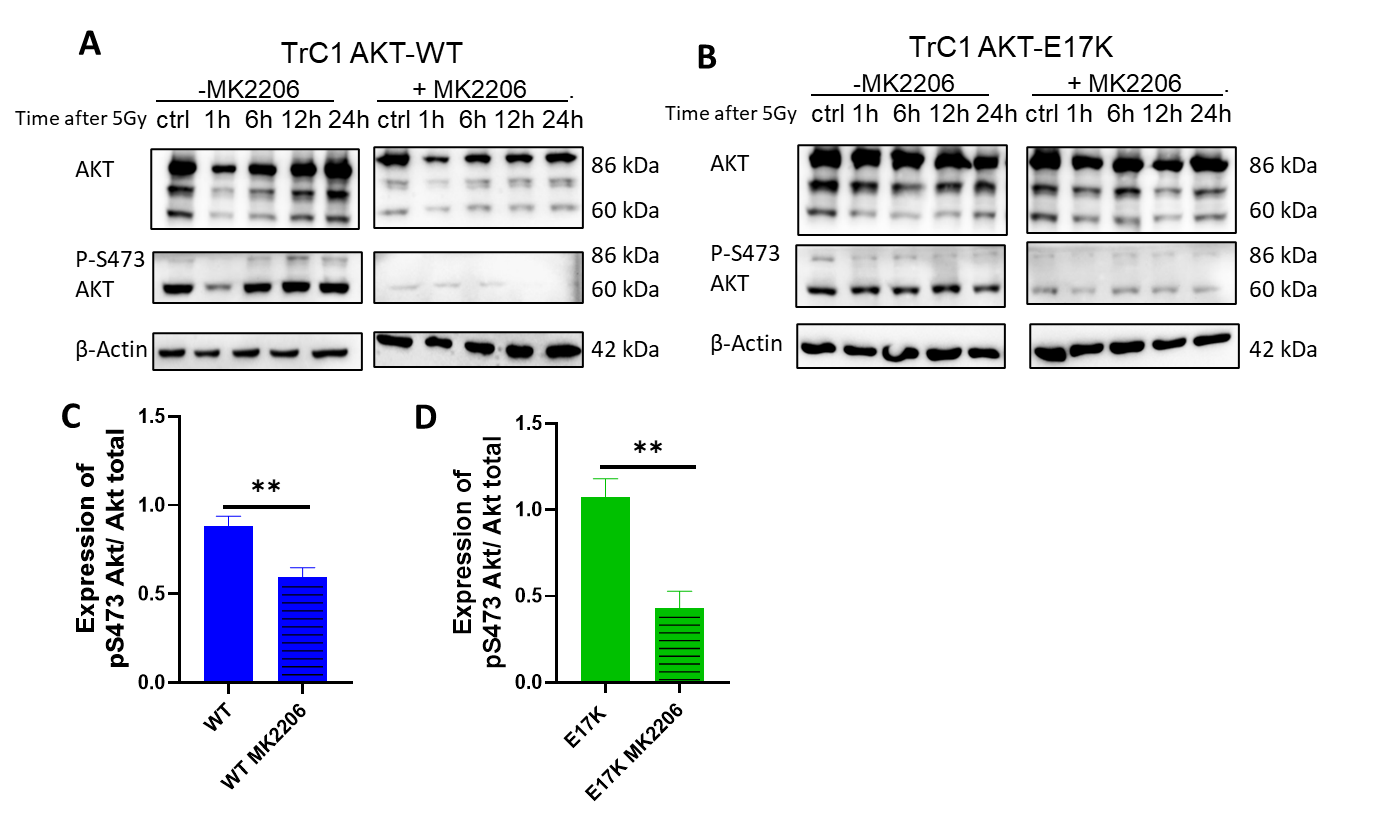


**Supplementary Figure 2: AKT inhibitor MK2206 abrogates AKT activation.** TrC1 AKT-E17K and AKT-WT cells were irradiated with 5 Gy and collected at implicated time points after IR as well as a 0 Gy control for the generation of lysates. Expression and phosphorylation level of AKT and phospho-Ser473 AKT, respectively, were determined by Western Blot analysis using β-Actin as a loading control in (A) for TrC1 AKT-WT cells and (B) in TrC1 AKT-E17K cells. Quantification of pS473-Akt amount normalized on total Akt and β-Actin are depicted for all time points in (C) for TrC1 AKT-WT and (D) for AKT-E17K cells showing control treatment compared to MK2206 treatment. Mean values and standard error of the mean (SEM) were scaled for data from at least 3 independent biological experiments with * p ≤0 .05, ** p ≤ 0.01, *** p ≤ 0.001, **** p ≤ 0.0001, ns p > 0.05 using an unpaired student’s t-test.


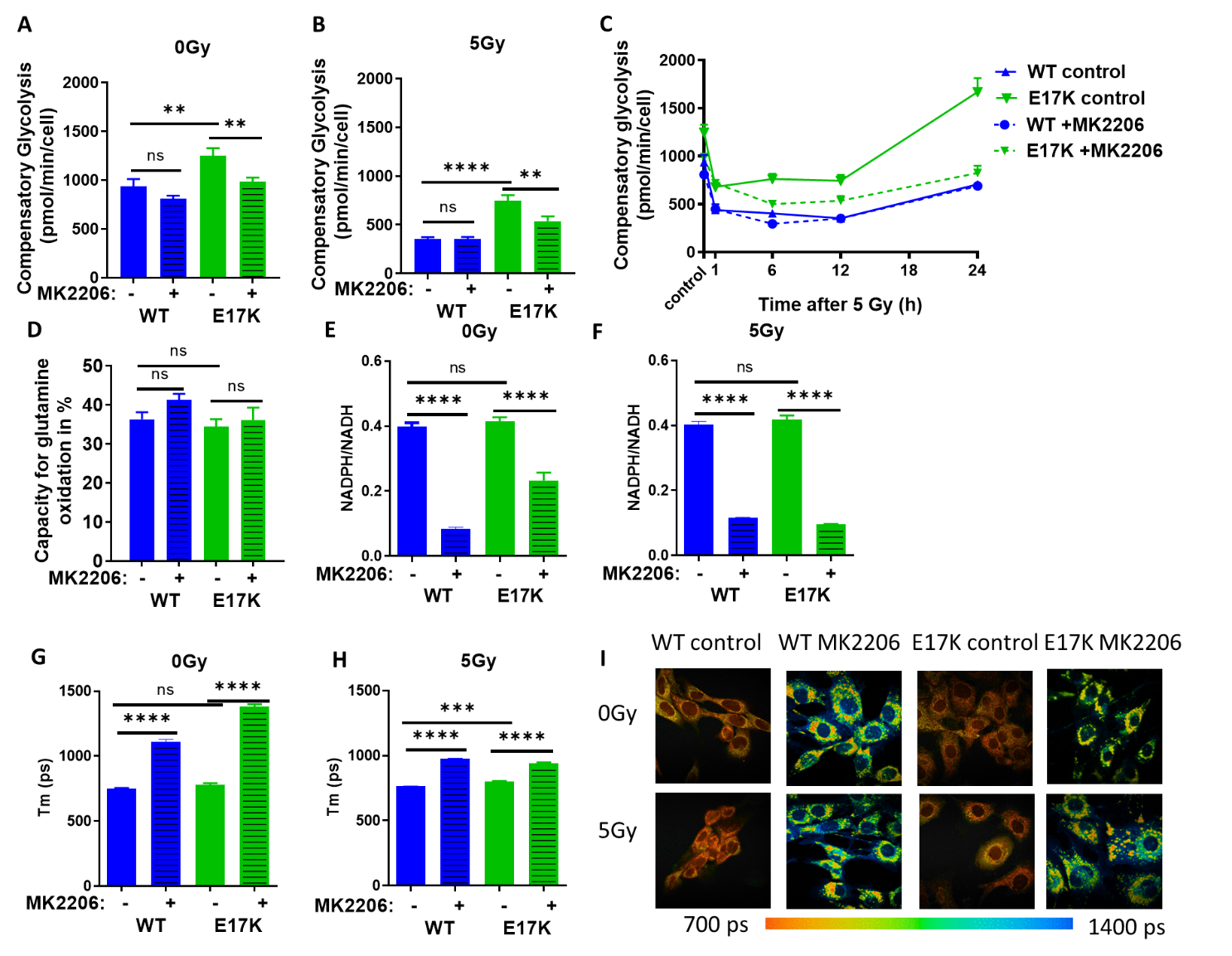


**Supplementary Figure 3: AKT-WT and AKT-E17K differ in their impact on the metabolic phenotype and the metabolic response of TrC1 cells to IR.** TrC1 AKT-WT and AKT-E17K cells were exposed to 0 Gy and 5 Gy with and without pretreatment for 1h with MK2206 (4 µM). Compensatory glycolysis (glycolytic rate upon OxPhos inhibition by rotenone and antimycin A) was determined by an extracellular flux assay (Seahorse technology) at baseline (0 Gy) and 24 h after exposure to 5 Gy (A-C). Values of compensatory glycolysis are shown in bar diagrams in (A, B), or as a time dependent regulation upon IR (C). Glutamine capacity (G) was analyzed using the fuel flex test (Seahorse technology). Mean values and standard error of the mean (SEM) were scaled for data from n = 8-16 wells from 2 independent biological experiments. Fluorescence lifetime imaging (FLIM) was used to determine NADPH/NADH ratio at baseline (0 Gy) and 24 h after exposure to 5 Gy (E-F). Additionally, bar charts of mean fluorescence lifetime (tm) of NADH and NADPH species measured in FLIM are displayed in (G) for 0 Gy and (H) for 5 Gy, and representative pictures of one representative experiment out of 3 (I). Mean values and standard error of the mean (SEM) were scaled for data from at least 3 independent biological experiments using one way ANOVA followed by multiple test. Stars depict with * p ≤ 0.05, ** p ≤ 0.01, *** p ≤ 0.001, **** p ≤ 0.0001, ns p > 0.05 .


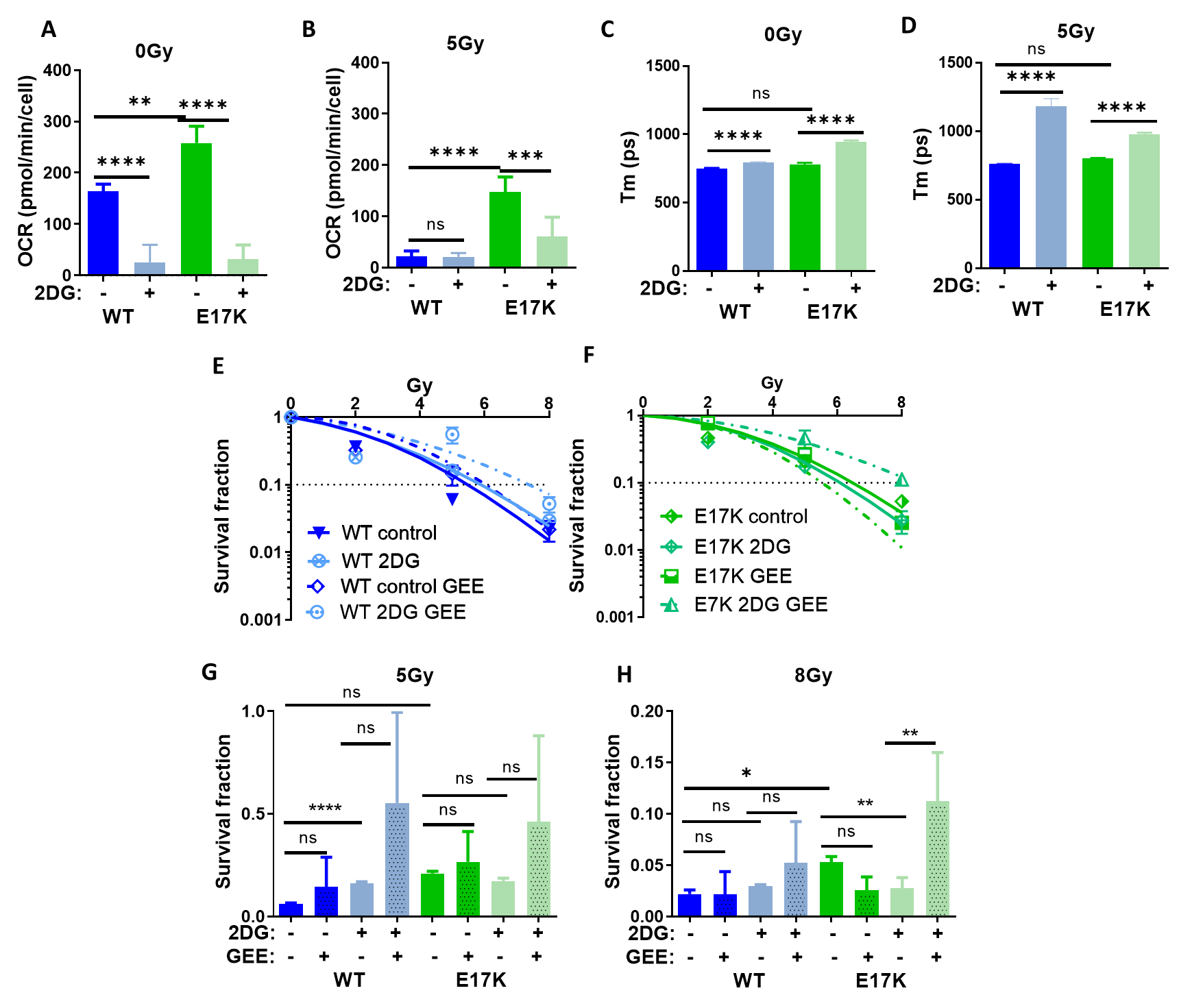


**Supplementary Figure 4: The effect of glycolysis inhibition on oxygen consumption rates (OCR) as well as short- and long-term survival upon irradiation is more pronounced in AKT-E17K TrC1 cells.** The effects of pre-treatment (1 h) with the hexokinase 2 inhibitor 2-deoxyglucose (2DG, 10 mM) and subsequent irradiation with 0 Gy or 5 Gy were measured in AKT-WT and AKT-E17K cells. (A, B) Mitochondrial respiration was determined by measuring OCR in an extracellular flux assay; bar diagrams for (A) 0 Gy and (B) 5 Gy 12 h after irradiation. Mean values and standard error of the mean (SEM) were scaled for data from n = 8-16 wells from minimum 2 independent biological experiments. (C, D) Mean fluorescence lifetime (tm) values were measured using FLIM. Mean values and standard error of the mean (SEM) were scaled for data from at least 3 independent biological experiments using one way ANOVA followed by multiple test. (E-I) Long term survival was determined by colony formation assay and using indirect plating upon treatment with 2DG (10 mM), without or with glutathione ethyl ester (GEE, 4 mM) compared to the respective controls; dose-dependent curves (E-F) and bar diagrams depict survival fractions upon irradiation with (G) 5 Gy or (H) 8 Gy, respectively. Mean values and standard error of the mean (SEM) were scaled for data from at least 3 independent biological experiments using two-way ANOVA followed by Tukey’s multiple comparison test. Stars depict * p ≤ 0.05, ** p ≤ 0.01, *** p ≤ 0.001, **** p ≤ 0.0001, ns p > 0.05.


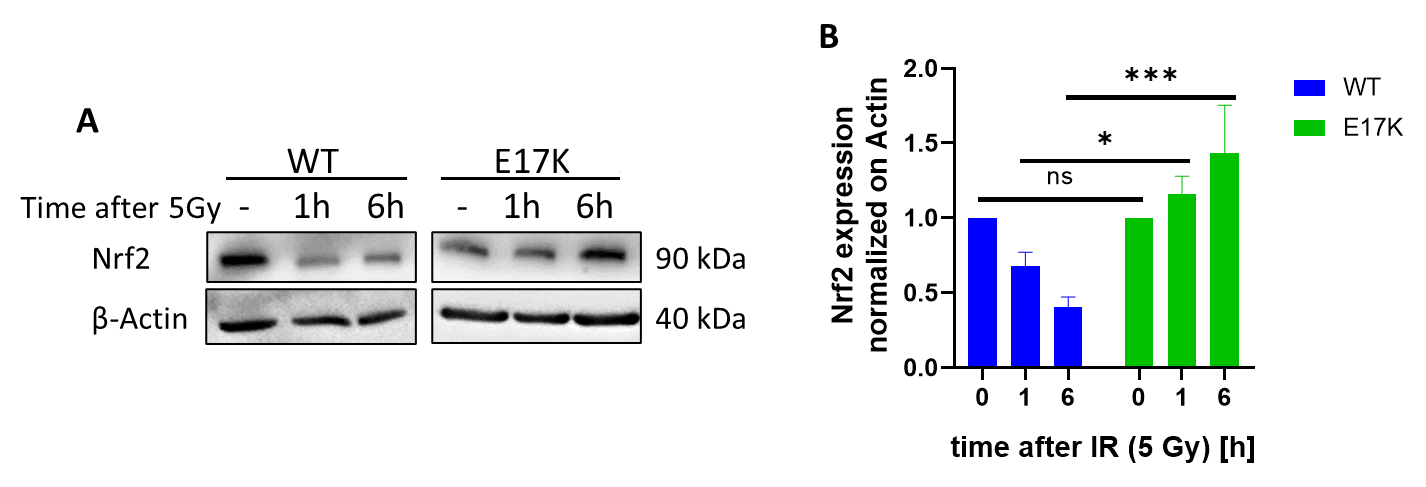


**Supplementary Figure 5: Differential regulation of nuclear factor erythroid 2-related factor 2 (Nrf2) expression levels in AKT-E17K and AKT-WT cells upon IR.** TrC1 AKT-E17K and AKT-WT cells were left untreated or irradiated with 5 Gy and collected without or at 1 h and 6 h after IR for the generation of lysates. Expression levels of Nrf2 were determined by Western Blot analysis using β-Actin as a loading control. Photomicrographs depict representative blots in (A) out of 3 independent experiments. Bar charts in (B) display mean values and standard error of the mean (SEM) of 3 independent biological experiments with * p ≤ 0.05, ** p ≤ 0.01, *** p ≤ 0.001, **** p ≤ 0.0001, ns p > 0.05 using two-way ANOVA followed by mixed effect analysis multiple comparison with Sidak’s correction.


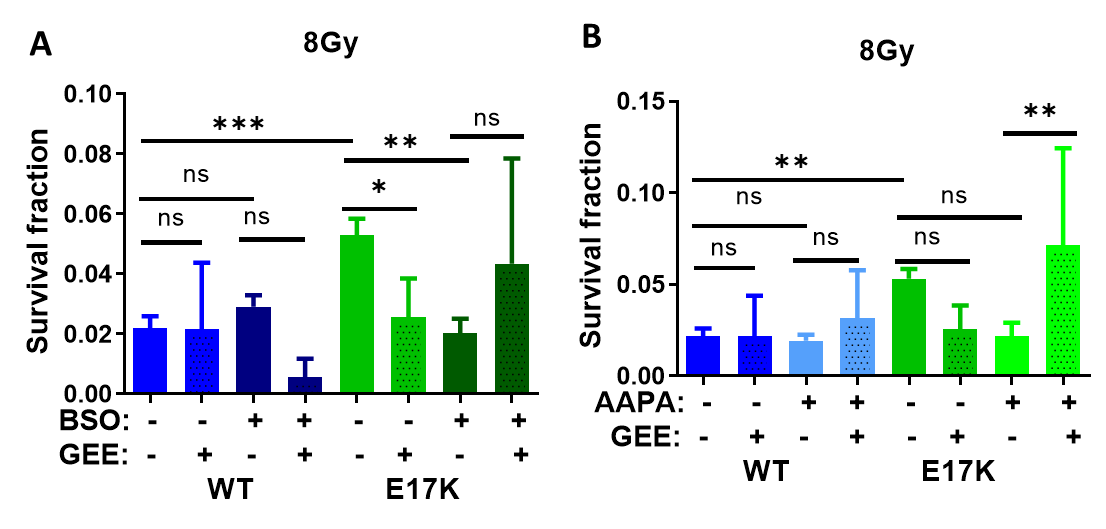


**Supplementary Figure 6: Addition of soluble GSH mimic in cells treated with inhibitors of GS or GR rescues GSH levels and restores enhanced radioresistance of TrC1 AKT-E17K cells.** TrC1 AKT-WT and AKT-E17K cells were treated with buthionine sulfoximine (BSO, 20 µM) without or with glutathione ethyl ester (GEE, 4 mM) (A) or with AAPA (4 µM) without or with glutathione ethyl ester (GEE, 4 mM) (B), and subsequently exposed to irradiation with 8 Gy, respectively. Bars depict mean values and standard error of the mean (SEM) from at least 3 independent biological experiments with * p ≤ 0.05, ** p ≤ 0.01, *** p ≤ 0.001, **** p ≤ 0.0001, ns p > 0.05 using two-way ANOVA followed by Tukey’s multiple comparison test.

**
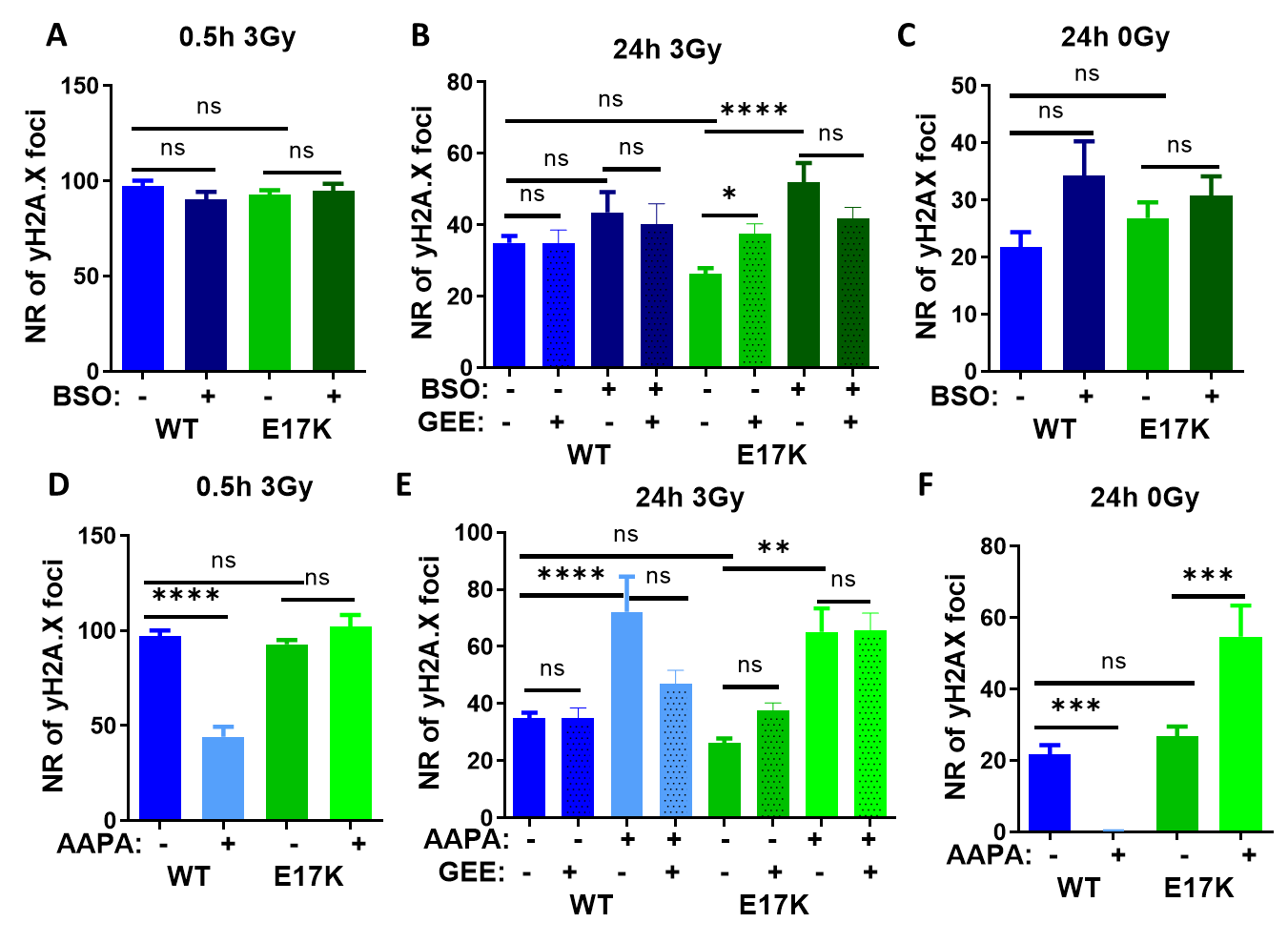
**

**Supplementary Figure 7: Effects of inhibition of glutathione synthase (GS), glutathione reductase (GR) or hexokinase 2 (HK2) on the amount of γH2A.X foci in TrC1 AKT-WT and AKT-E17K cells.** TrC1 AKT-WT and AKT-E17K cells were treated with BSO (20 µM) (A) or with AAPA (4 µM) (D) 1 h prior IR with 3 Gy and numbers of γH2A.X foci were determined by immunofluorescence 30 min after IR treatment or 24 h after IR treatment without and with additional treatment with glutathione ethyl ester (GEE, 4 mM) (B, E). Treatment controls 24 h after inhibitor treatment but without IR are depicted in (C, F). Mean values and standard error of the mean (SEM) were scaled for data from at least 3 independent biological experiments with * p ≤ 0.05, ** p ≤ 0.01, *** p ≤ 0.001, **** p ≤ 0.0001, ns p > 0.05 using one way ANOVA followed by multiple test.
